# Nano chitosan peptide as a potential therapeutic carrier for retinal delivery to treat age-related macular degeneration

**Published:** 2012-09-03

**Authors:** Melamangalam S. Jayaraman, Dhruba J. Bharali, Thangirala Sudha, Shaker A. Mousa

**Affiliations:** Pharmaceutical Research Institute at Albany College of Pharmacy and Health Sciences, One Discovery Drive, Rensselaer, NY

## Abstract

**Purpose:**

We describe the synthesis and use of an efficient nano carrier molecule for retinal delivery of a nano chitosan peptide that has potential application for treating age-related macular degeneration (AMD). We chose serine-threonine-tyrosine as the peptide sequence because it is well known to act as a transduction signaling agent within and between retinal pigmented epithelium cells.

**Methods:**

A nanoformulation of a water-soluble chitosan conjugated with a peptide (serine-threonine-tyrosine) was synthesized by a method developed in our laboratory and characterized with dynamic light scattering, zeta potential, transmission electron microscopy, nuclear magnetic resonance, and Fourier transform infrared spectroscopy. The in vitro efficacy of the formulation was evaluated in retinal cells with confocal microscopy by studying the formulation’s action on tyrosine kinase activity.

**Results:**

The conjugated nano chitosan peptide showed evidence of tyrosine kinase activity as seen by fluorescent signals under confocal microscopy, while nano chitosan or peptide alone did not show such activity.

**Conclusions:**

Conjugated nano chitosan peptide may promote binding and engulfment. This molecule is an excellent carrier for retinal drug delivery and has the potential to treat age-related macular degeneration.

## Introduction

Age-related macular degeneration (AMD) is the leading cause of blindness in the US, affecting more than 1.75 million people [1], and it is predicted that 3 million Americans will be affected by AMD by 2020 [2]. AMD is a disease mainly caused by pathological changes due to the disruption of the usual function of retinal pigmented epithelium (RPE) cells by oxidative stress [3,4]. RPE forms part of the blood–retinal barrier with a single layer of pigmented cells and acts as a deterrent to the delivery of any external active substances such as a drug. RPE plays an important role in the visual cycle, especially recycling spent photoreceptor outer segments (POS) by a process called phagocytosis. Failure of phagocytosis leads to accumulation of POS, and chronic accumulation leads to the blockage of vision. Despite relentless effort by the scientific community in the past several decades, AMD remains a progressive disease with no complete cure. Current Federal Drug Administration-approved treatment modalities are expensive, rely on invasive techniques, and are not capable of completely restoring vision.

AMD is classified into two categories: dry type and wet type (neovascular). Both types eventually lead to vision loss. The dry type is more common than the wet type, with about 85% to 90% of patients with AMD diagnosed with dry type AMD. Only dietary supplements are available to treat dry type AMD. Clinical trials with Othera Pharmaceuticals’ antioxidant eye drops OT551 for dry type showed promise in phase 1 trials, but failed to demonstrate any benefit greater than from placebo in phase 2 trials [5]. Recently, the Federal Drug Administration approved “Center Sight”—a tiny implantable miniature telescope (IMT). When surgically implanted in the cornea, an IMT enables magnification of an image on the intact peripheral retina. An IMT can be used for wet and dry end stage AMD, although the procedure is expensive [6,7]. In wet type AMD, photodynamic therapy was the most common treatment to preserve existing vision, but relapse occurred in the majority of cases [8-10]. Antivascular endothelial growth factor treatments are now the most common treatment for wet type AMD, yet drugs such as Lucentis and Macugen have complications that include endophthalmitis, increased intraocular pressure, traumatic cataract, detached retina, and stroke [11-14]. Current trials with small interfering RNA products show promise but lack efficacy [15]. Therefore, it is imperative to look for alternative noninvasive, user-friendly technologies that have the potential to overcome these problems to treat dry and wet AMD efficiently, and at lower cost.

The emergence of nanotechnology may have a profound effect on ocular biomedical applications, especially delivery of drugs to the posterior of the eye via nano carriers [16-18]. Ideally, nano carriers should elude body defense mechanisms, thus evading biologic and physiologic barriers. Nano carriers must be excellent carriers for the active moiety and allow controlled, sustained release of drugs. Nano carriers not only protect the incorporated active moiety but also effectively deliver it to the specific action site by targeted delivery [19,20]. Increase in cell-specific targeted delivery to the posterior of the eye depends on judicious manipulation of the size and surface charge of suitable nano carriers, and hence increases the permeability. Nano carriers, with their unique small size, are less inflammatory and devoid of irritable side effects as opposed to regular formulations. Nano carriers should also be biodegradable to avoid accumulation in the eye.

Chitosan, a water-soluble polysaccharide extracted from crab shell, has excellent properties for ideal ocular delivery of drugs, not only meeting these criteria but also proving to be superior compared to other polymers used for ocular delivery [21,22]. Conjugated chitosan peptide mediates cell adhesion, attachment, and spreading, similar to integrins [23]. A signal peptide is a transit peptide of 3–60 amino acids that targets proteins in organelles such as mitochondria, and acts by simple propagation of signal to ligand-receptor interactions. Signal peptides are rapidly degraded by the presence of degrading enzymes, rendering them poor bioavailable substances when introduced exogenously. Due to the mucoadhesive properties of chitosan, incorporating a signal peptide drug in a nano chitosan particle increases the drug’s stability and residence time, and thus can increase the bioavailability of the drug at the desired site [24,25]. This leads to the revitalization of signal transduction with an exogenous signal peptide that can reset cellular events. Signal peptides thus can be delivered to induce or stimulate cell functions that were previously dysfunctional.

Normal RPE phagocytosis is the process of recycling POS, and includes binding, engulfment, and internalization of POS. Binding and engulfment occur in the presence of integrin αvβ5 and focal adhesion kinase, and then internalization can occur as initiated by the tyrosine kinase enzyme MerTK. This enzyme is stimulated by phosphorylation of tyrosine [26]. Defective phagocytosis may be due to defective binding of POS because of a diminished amount or lack of αvβ5 [27,28]. Defective phagocytosis may also be due to defective internalization because of non-stimulation of MerTK, although non-specific phagocytosis (not involving MerTK) may occur through alternative pathways such as macrophages. Facilitating the stimulation of MerTK and the internalization process using a signal peptide containing tyrosine may result in a signal cascade and streamline phagocytosis.

We hypothesize that delivering tyrosine to RPE with a vehicle such as a nano chitosan peptide may significantly enhance phagocytosis by stimulating MerTK. We made a nano carrier by conjugating the signal peptide serine-threonine-tyrosine (ser-thr-tyr) to water-soluble, low-molecular-weight chitosan. Ser-thr-tyr functions as a transduction signaling agent within and between RPE cells [29]. Once within the cell, ser-thr-tyr may be cleaved and phosphorylated [30]. It is ideal to exploit this phenomenon so tyrosine phosphorylation can stimulate and activate MerTK for internalizing POS. Serine-threonine may interact directly with the serine-threonine kinase receptor to inhibit preapoptotic cells from apoptosis. The experiment was conducted on RPE cells by using the tyrosine kinase blocker Lavendustin-A [31] and tyrosine antibodies, and observing tyrosine kinase activity under confocal microscopy.

## Methods

### Materials

Low-molecular-weight, water-soluble chitosan (chitosan oligosaccharide) was purchased from Kittolife (Seoul, South Korea). Di-tert-butyl carbonate serine-threonine-tyrosine (TBOC ser-thr-tyr) was custom-synthesized by CHI Scientific (Maynard, MA). Lavendustin-A (tyrosine kinase blocker) was purchased from Tocris Bioscience (Ellisville, MO). Mouse antiphosphate 4G10 platinum-unconjugated was purchased from Millipore (Billerica, MA). N-(3-dimethylaminopropyl)-N’-ethylcarbodiimide HCl (EDAC), pentasodium tripolyphosphate (TPP), dimethyl sulfoxide (DMSO), trifluoroacetic acid (TFA), and dichloromethane (CH_2_Cl_2_) were purchased from Sigma Aldrich (St. Louis, MO). An immortalized human RPE cell line was purchased from ATCC (ARPE-19 cells, Manassas, VA), and other cell components were purchased from Invitrogen Life Technologies (Carlsbad, CA). Tyrosine kinase was obtained from Millipore, and secondary antibodies were from Sigma Aldrich.

### Synthesis of chitosan peptide

**Figure 1:**
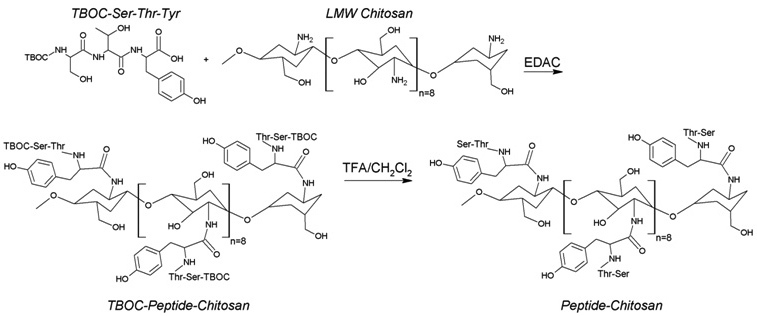
Chemical reaction showing the peptide serine-threonine-tyrosine (ser-thr-tyr) protected with di-tert-butyl carbonate (TBOC) and conjugation of TBOC-ser-thr-tyr to chitosan. The final step is to remove the TBOC with trifluoroacetic acid/dichloromethane (TFA/CH_2_Cl_2_). LMW, low molecular weight; EDAC, N-(3-dimethylaminopropyl)-N’-ethylcarbodiimide HCl.

The scheme for the overall synthesis is shown in Figure 1. TBOC is widely used to synthesize peptides and to protect amino groups from chemical modification. In this experiment, we used ser-thr-tyr as the peptide, and protected the amino group at the serine end with TBOC before conjugation to chitosan. To synthesize TBOC-ser-thr-tyr chitosan, 12 mg of chitosan was dissolved in about 1 ml of deionized water, and then 6 mg of TBOC ser-thr-tyr dissolved in 12 µl of DMSO was added with constant magnetic stirring (about 1000 rpm). To this mixture, 12 mg of EDAC was added, and the mixture was stirred for 3 h. After stirring, the impurities and unreacted materials were removed by continuous dialysis against distilled water with an RC Dialysis Bag (cutoff 5000–8000 MW, Spectrum Laboratories, Inc., Rancho Dominguez, CA) for at least 24 h. The entire dialyzed solution was then lyophilized to obtain TBOC-ser-thr-tyr chitosan in powdered form. To remove TBOC from ser-thr-tyr chitosan, 1.2 ml of TFA:CH_2_Cl_2_ (1:3) was added to the TBOC-ser-thr-tyr chitosan powder and stirred for 3 h at 25 °C, and then the mixture was evaporated using a Buchi-Rotovapor-205 rotary evaporator (Buchi Corp., New Castle, DE). Residual TFA was removed by passing a steady stream of nitrogen over it. It was finally dried in a vacuum oven. The final product was ser-thr-tyr chitosan, and this was used for further synthesis of nanoparticles as described below.

### Synthesis of chitosan peptide nanoparticles

Chitosan nanoparticles conjugating the peptide were obtained using ionic gelation of chitosan with TPP anions [32,33]. Thus in a typical experiment, to 6 ml of chitosan peptide of concentration 1.5 mg/ml in deionized water, 1 ml of TPP solution (1 mg/ml deionized water) was added drop by drop under constant magnetic stirring (1000 rpm) at room temperature. The entire solution was then vortexed intermittently and sonicated for 3 s (Branson sonifier Model 150, 120 MHz, Branson Ultrasonics Corp., Danbury, CT). The entire solution was stirred for an additional 1 h, and filtered through a 0.45 micron Millipore sterile filter. The solution was then lyophilized to obtain the final nanoformulation in powdered form. This powered form of the nanoparticles containing the peptide was redispersed for further analysis and efficacy study. Void chitosan nanoparticles (without peptide) were synthesized using a similar method with an equivalent amount of chitosan as the starting material instead of the chitosan peptide. To check the reproducibility of the synthesis, 24 batches of the nanoparticles were made and analyzed by dynamic light scattering (described below).

### Size measurement with dynamic light scattering

The size distribution of void chitosan nanoparticles and chitosan peptide nanoparticles in aqueous dispersion was determined by placing 1 ml of a sample in a four-sided, clear, plastic cuvette in a Malvern Zeta sizer (Malvern Instrumentation Co, Westborough, MA). Analysis was conducted directly at 25 °C.

### Zeta potential measurement

The zeta potential of nanoparticles is the charge that develops near the surface of the particles; the zeta potential is a fundamental parameter of colloidal dispersion and indicates the stability of a nanoformulation. The zeta potential is expressed in mV, and typically, values above 30 mV (absolute value) are considered physically stable. Below 20 mV (absolute value), there is partial stability, and below 5 mV (absolute value) rapid aggregation occurs. The zeta potential of the void chitosan and chitosan peptide nanoparticles in aqueous dispersion was determined by using the same Malvern zeta sizer instrument. Around 750 µl of a resuspended sample were placed in a disposable capillary zeta potential cell, available for the zeta sizer nano series. The measurement was performed at 25 °C.

### Transmission electron microscopy

The size and morphology of the chitosan peptide nanoparticles were examined by using a JEM-100CX transmission electron microscope (JEOL, Inc., Peabody, MA). One drop of the resuspended (from lyophilized powder) nanoparticles in deionized water was mounted on a thin film of amorphous carbon deposited on a copper grid (300 mesh). It was then dried in clean conditions, stained with 1% uranyl acetate, and the grid was examined directly under the TEM. The pictures of the samples were captured using a camera attached to the instrument.

### Nuclear magnetic resonance and Fourier transform infrared spectroscopy

The structure of the chitosan peptide nanoparticles was characterized with proton nuclear magnetic resonance (NMR) at 500 MHz (Bruker Spectrospin, Billerica, MA), and Fourier transform infrared (FTIR) spectroscopy (Model AVATAR 330, Thermo Electric Corporation, Waltham, MA). The NMR solvent was deuterated DMSO. For FTIR, the glass plate was cleaned in between samples and standardized with blank readings, and the dry powders of nano chitosan, peptide, and chitosan peptide nanoparticles were placed on the glass plate, and the spectra obtained.

### In vitro efficacy evaluation

RPE cells were maintained in Dulbecco’s modified Eagle medium (DMEM) supplemented with 10% fetal bovine serum at 37 °C with 5% CO_2_. Cells growing in chamber slides were preincubated with Lavendustin-A for 3 h before being treated with the test compounds (nano chitosan, nano chitosan peptide, and peptide). After 1 h, cells were fixed in 4% paraformaldehyde, and tyrosine kinase activity was analyzed using antiphosphotyrosine antibodies. The primary antibody was detected with Alexa Fluor 488, and cytoskeletons were stained with rhodamine-phalloidin and the nuclei with 4',6-diamidino-2-phenylindole (DAPI).

### Confocal imaging

We used a Leica TCS SP5 confocal microscope (Buffalo Grove, IL). Tyrosine kinase signals (green) were detected at 494 nm excitation and 518 nm emission, rhodamine-phalloidin stain signals were detected at 542 nm excitation and 565 nm emission, and DAPI signals were detected at 345 nm excitation and 448 nm emission. Pictures were acquired at 40× magnification. The intensity of the signals was measured using the software Leica Application Suite Advanced Fluorescence (LAS AF). Images for 6 fields per sample were analyzed for fluorescence signal intensity. The error bars indicate standard errors of the mean (SEM).

## Results

### Characterization of chitosan void nanoparticles and chitosan peptide nanoparticles

**Figure 2:**
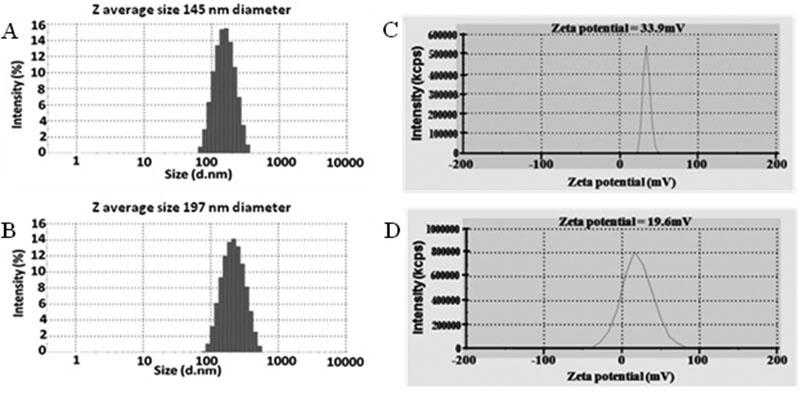
Characterization of nanoparticles with dynamic light scattering and zeta potential measurements. Dynamic light scattering data show the size of (**A**) the chitosan void nanoparticles and (**B**) the chitosan peptide nanoparticles. The zeta potential is shown for (**C**) the chitosan void nanoparticles and (**D**) the chitosan peptide nanoparticles. The polydispersity index for both was around 15%.

**Figure 3:**
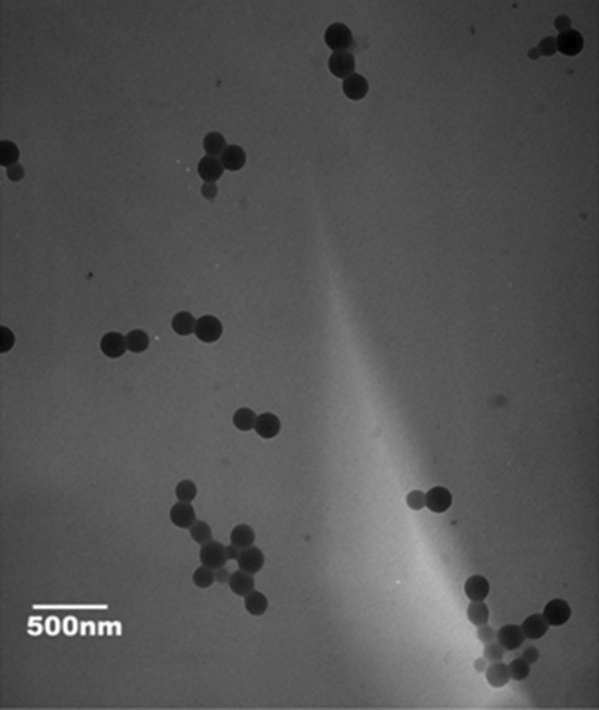
Characterization of nanoparticles with transmission electron microscopy shows the size and morphology of the chitozan peptide nanoparticles.

Figure 2A,B show the dynamic light scattering (DLS) data of the chitosan void nanoparticles and chitosan peptide nanoparticles. The average size of the void nanoparticles was about 150 nm in diameter, but the chitosan peptide nanoparticles were larger, at about 200 nm in diameter. The zeta potential (Figure 2C,D) revealed that the chitosan void nanoparticles were much higher (about 34 mV), while the chitosan peptide nanoparticles were about 20 mV. The polydispersity index (PDI) for both was around 15%. The transmission electron microscopy (TEM) image (Figure 3) further supports the evidence of the size determined with DLS. In addition, from Figure 3 it is clear that the nanoparticles were spherical.

### Nuclear magnetic resonance and Fourier transform infrared spectra of peptide, chitosan, and chitosan peptide nanoparticles

**Figure 4:**
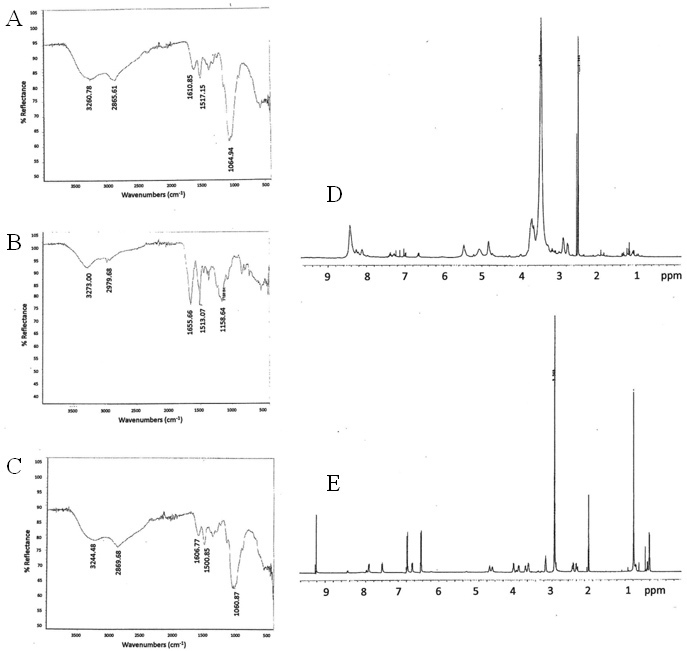
Characterization of nanoparticles with nuclear magnetic resonance and Fourier transform infrared spectroscopy. The Fourier transform infrared spectra shown are (**A**) chitosan peptide nanoparticles, (**B**) ser-thr-tyr peptide, and (**C**) chitosan. The nuclear magnetic resonance spectra shown are (**D**) the chitosan peptide nanoparticles and (**E**) the ser-thr-tyr peptide.

NMR and FTIR spectra of the signal peptide, chitosan, and chitosan peptide nanoparticles are shown in Figure 4. The FTIR spectrum of the chitosan peptide nanoparticles (Figure 4A) does not contain the tyrosine C=O stretch as seen in the peptide spectrum (Figure 4B) at 1655 cm^−1^, supporting the conjugation of chitosan (FTIR spectrum shown in Figure 4C) with the peptide via amide formation. We followed up the FTIR characterization by collecting NMR spectra, where the ^1^H chemical shifts play a role in identifying functional groups. Examination of the NMR spectra of the chitosan peptide nanoparticles versus peptide showed that in the chitosan peptide nanoparticles spectrum there were broad, downfield-shifted signals between 8.0 and 8.6 ppm (Figure 4D), compared to the sharp tyrosine aromatic ring proton signals in the peptide spectrum between 6.6 and 7.0 ppm (Figure 4E). The 8.0 to 8.6 ppm signals are expected to be the tyrosine aromatic proton signals. The chitosan peptide nanoparticles’ NMR signals in general were broad compared to the peptide signals due to the bulk of the chitosan polymer, and the downfield shift of the tyrosine aromatic signals was expected due to the influence of the electronegative N, O atoms of the amide group formed with tyrosine in the conjugated chitosan-peptide.

### Efficacy test with confocal imaging

**Figure 5:**
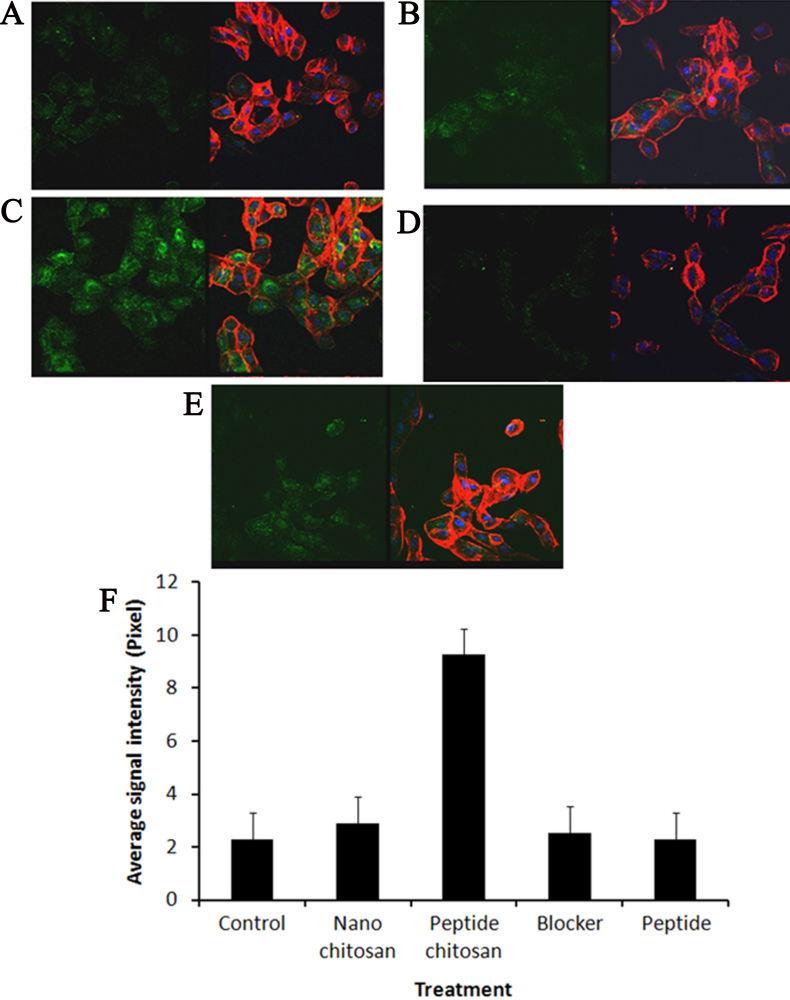
Study of tyrosine kinase activity in RPE cells with confocal microscopy. For each pair of confocal images, the left panel shows the phosphotyrosine signals, and the right panel shows the overlay of protein, cytoskeleton, and nuclear stains (**A**) control RPE cells without any treatment, (**B**) treatment with nano chitosan (10 μM) for 5 h had no effect on tyrosine kinase expression, similar to the control, (**C**) peptide conjugated with nano chitosan (10 μM) increased the expression of tyrosine kinase proteins, (**D**) pretreatment for 1 h with tyrosine kinase blocker [Lavendustin-A (50 μM)] clearly suppressed protein expression, (**E**) incubation with peptide (10 μM) alone had no effect on the protein. Green: anti-phosphotyrosine antibody (Alexa Fluor 488); red: actin cytoskeleton (rhodamine-phalloidin stain); blue: nucleus (DAPI), (**F**) quantification of tyrosine kinase signals under different treatment conditions as described for **A** to **E** above.

For proof of the concept that chitosan peptide nanoparticles can be used as an efficient delivery vehicle, we used RPE cells to view their uptake and their action on tyrosine kinase activity. Chitosan peptide nanoparticles increased tyrosine kinase activity as evidenced by strong fluorescent signals seen with confocal microscopy (Figure 5). Lavendustin-A, a specific tyrosine kinase blocker [31], completely inhibited the expression of tyrosine kinase even in the presence of chitosan peptide nanoparticles. Neither nano chitosan nor peptide alone had any effect on tyrosine kinase. The intensities of nano chitosan and peptide alone were comparable to the control.

## Discussion

A method for synthesizing chitosan nanoparticles conjugating a small peptide, ser-thr-tyr, was developed and optimized in terms of size and zeta potential in our laboratory. Our technique has proven to be effective in preventing dimerization by blocking free -NH_2_ groups in the serine moiety of the signaling protein. Techniques such as NMR and FTIR can be used to effectively follow the reaction, even though the conjugation was in nanosized form.

Though the DLS data show a small increase in size of the nanoparticles when conjugated to the peptide, this increase may be due to the increase in the polymer size by the addition of the peptide to the chitosan backbone. Since chitosan polymers contain numerous free -NH_2_ groups, there will be multiple ser-thr-tyr peptide conjugates on the same polymer. This increases the bulkiness of the polymer, evidenced by the increase in the size of the nanoparticles. Though the zeta potential data show that void and chitosan peptide nanoparticles are positively charged, the zeta potential significantly decreased upon conjugation to the peptide. This was expected due to the added bulk of the peptide. In void nanoparticles, most of the positively charged -NH_2_ groups are free, and hence lead to a high positive zeta potential. When chitosan is conjugated with the peptide, many of the terminal free -NH_2_ react to form amide bonds between chitosan and ser-thr-tyr. This lowers the cationic charges by the absence of free -NH_2_ groups, and hence lowers the zeta potential. Improved methods of preparation might improve the stability of these chitosan peptide nanoparticles in solution.

Water-soluble, low-molecular-weight chitosan was the best choice as the building block of the nanoparticles owing to its excellent properties like mucoadhesiveness, biocompatibility, and a safe history. In addition, the free -NH_2_ groups available in chitosan polymer can be used for conjugation in a relatively less harsh chemical reaction. Additionally, the choice of water-soluble chitosan has the edge over its counterpart, water-insoluble chitosan, because of its antiangiogenic properties, an important prerequisite for ocular delivery. It has been hypothesized that inhibition of heparanase activity by water-soluble, low-molecular-weight chitosan is due to its antiangiogenic properties (although chitosan adheres to tissues superficially, it is not involved in cell attachment) [34]. Researchers have reported that chitosan, when conjugated to peptide, can behave like an extracellular matrix mimetic [35], making it an excellent scaffold matrix for cell attachment to integrins, glycosylated transmembrane adhesive receptors, and syndecans. The antiangiogenic property of chitosan enables it to act as a free carrier to retinal cells rather than recruit neovascular structures. Various chitosan conjugated peptide preparations have shown cell attachment properties at various sites; hence, we tested our preparation at the retinal level.

We chose the three amino acid combination ser-thr-tyr because it constitutes a signal peptide. Serine threonine kinases, with phosphorylation, can act as signal peptide as seen in alveolar macrophages [36]. Similar signaling effects may be seen in RPE. Serine-threonine kinase phosphorylates serine-threonine to preserve mitochondrial function and prevent mitochondrial swelling of mitochondrial terminal pore and cell death [37]. Protein kinase B (PKB), a serine-threonine kinase, blocks apoptotic process and promotes cell survival, acting as a key signaling protein for the cell survival pathway [38]. Our tests revealed that when blocking RPE cells with Lavendustin-A, a specific tyrosine kinase blocker, and then fixing the cells with tyrosine antibodies, only nano chitosan peptide, but not nano chitosan or peptide alone, enabled focal adhesion kinase-integrin adherence, and binding and engulfment. POS can also be internalized by the phosphorylation of the tyrosine of MerTK, promoting phagocytosis. Nano chitosan peptide thus is an excellent, promising molecule for stimulating and promoting RPE phagocytosis. In future experiments we plan to test different peptide sequences in comparison to ser-thr-tyr to determine if ser-thr-tyr is the best choice of signal peptide for this chitosan peptide nano carrier.

One aspect of AMD is age-related deterioration of melanin pigments in RPE, which leads to reduced antioxidant activity [39]. Tyrosine-derived synthetic melanin increased melanin aggregation in cultured RPE cells, and increased blue light protection, making it less susceptible to apoptosis [40]. Tyrosinase converts tyrosine to melanin on melanocytes by a process called melanogenesis. Despite the classical hypothesis of melanogenesis in RPE at the embryonic stage, in vitro experiments conducted in cultured adult RPE cells showed evidence of tyrosinase expression and its enzyme activity by phagocytosis of POS [41]. We anticipate that by delivering tyrosine to RPE cells via a conjugated nano chitosan peptide, an increase in melanin aggregation is possible. This may increase the antioxidant activity of melanin, reducing oxidative stress. Future experiments are needed to test this beneficial effect.

We have successfully synthesized and optimized therapeutic chitosan nanoparticles conjugated to a ser-thr-tyr signal peptide. Our data show that this nano chitosan peptide is an excellent carrier molecule that has the potential to promote RPE binding, leading to the engulfment process necessary for phagocytosis. Additionally, the advantage of this nano-sized peptide carrier is its prospective for ocular delivery of a mitochondrial antioxidant moiety that can reach the target site within the cell for effective pharmacological action. This may alleviate mitochondrial stress, retard the degenerative process, and normalize phagocytosis. Thus, our experiments show strong evidence that a nano chitosan peptide has the potential to help recycle POS through RPE phagocytosis, and therefore will be a better choice over conventional delivery of drugs and antioxidants used in current treatments for AMD. Though further studies may be needed to evaluate the role of mitochondrial antioxidants delivered through carrier molecules in relieving or reversing oxidative stress, certainly our positive results with a nano chitosan peptide may lead the way forward toward effective treatment of AMD.
